# Fluorescent Holographic Fringes with a Surface Relief Structure Based on Merocyanine Aggregation Driven by Blue-violet Laser

**DOI:** 10.1038/s41598-018-22202-2

**Published:** 2018-02-28

**Authors:** Ruiya Ji, Shencheng Fu, Xintong Zhang, Xiuxiu Han, Shuangyan Liu, Xiuli Wang, Yichun Liu

**Affiliations:** 1 Center for Advanced Optoelectronic Functional Material Research, Northeast Normal University and Key Laboratory of UV-Emitting Materials and Technology (Northeast Normal University), Ministry of Education, Changchun, 130024 P. R. China; 2 School of Life Science, Northeast Normal University, Changchun, 130024 P. R. China

## Abstract

Stability and integration are the goals for developing photonic devices. Spirooxazines have the property of photoinduced merocyanine-aggregation in polymer matrix, which can be applied to fluorescence emission and stable information storage. Although visible light coherent radiation with UV-assist has been used to achieve polarization-modulated holographic memory in spirooxazine doped PMMA films, the complexity of optical systems is increased and the aggregation ability of merocyanine is decreased. Here, we report that fluorescent holographic gratings with a surface relief structure can be inscribed in the film *via* sole irradiation of 403.4 nm. Time-dependent photo-anisotropy and holographic dynamics were both investigated with different power densities of the near-UV laser. The non-exponential photokinetics was explained by the sequential formation of mono- and aggregate-merocyanine molecules. The appearance of merocyanine aggregates is found to be beneficial to the long-term holographic memory with fluorescent emission. This work provides a research strategy for the integrity of storage, display and micro-fabrication of organic functional-devices.

## Introduction

High-performance optoelectronic integrated devices are pursued for the current information technology^1–6^. Organic photochromic materials have advantages of low-cost, flexibility, large-area film-forming, as well as the controlled physical parameters of absorption coefficient, refractive index and dielectric constant. Besides, information transport^7^, display^8–10^, storage^3,11–13^ and micro-fabrication^14,15^ have been realized in organic photochromic materials, which provide the possibility of their application in integrate photonics^16^.

Spirooxazine (SO) is one of the interesting families of organic molecules with photochromic property. They exhibit much better performance in photo-response, environment-stability and fatigue-resistance, compared with conventional photochromic materials, such as spiropyrans^17^. The colorless SO molecules undergo a heterolytic ring cleavage upon (near) UV irradiation, producing colored form of merocyanine (MC). Meanwhile, the MC molecules are able to be transformed into the colorless close-structure under the visible light irradiation or thermal treatment. Alternately, further implementation to excite the MC state with the high frequency light will result in fluorescent emission in visible region, which provides possibility for biological fluorescent labeling, such as selectively highlighting cells, organelles, or proteins^18^. Besides, the color-reversible film can also be applied in tunable photochromism^19^, distributed feedback (DFB) laser^20^, optical switch^21^ and 3D memory^22^. Especially, polarization holographic storage in SO doped polymers was realized by anisotropic molecule transformation in the visible-laser recording assisted with UV-irradiation, which put the SO-MC system forward to the applications of polarization conversion and high-density information memory^23–31^. Recently, bi-photonic polarization holographic recording was also realized in the film using two visible lasers with different wavelengths^32–34^. However, little attention was given to mono-color induced reaction processes in SO-MC system, which can not only resolve the complexity of optical systems but also help for the functional integration of the potential molecule device.

In this paper, the high-efficient and stable florescent holographic gratings with a surface relief structure were inscribed by coherent irradiations from a blue-violet laser with different power densities in SO-doped polymers. The holographic kinetics presents an interesting oscillation behavior, which can be explained by the sequential formation of mono-merocyanine (MC_mono_) and aggregate-merocyanine (MC_agg_). Benefiting from the stability of MC_agg_, long-term hologram memory and fluorescence emission were both realized in the photochromic film.

## Materials and Methods

### Materials and film preparation

1,3-Dihydro-1,3,3-trimethylspiro [2H-indole-2,3′-[3 H] phenanthr[9,10-b](1,4)oxazine (SO) with the purity of 98% and poly(methy methacrylate) (PMMA) were purchased from Aldrich. Molecular weight of PMMA is 400000~550000. SO and PMMA (weight ratio of 1:5) were full of dissolved in chloroform to obtain a mixed solution with the solution concentration of 10 mg/ml. After stirring for 30 min by a mixing machine, the solution of 200 μL was drop-coated on a clean slide glass to produce enough thick samples for effective holographic recording. A watch glass was buckled to the sample so that the evaporation rate can be controlled well. The photochromic film was obtained after the solvent evaporated at room temperature (300 K) for 12 h in the dark. The sample surface morphology (surface height change of less than 150 nm) and the resultant film thickness (~2.38 μm), were measured by a step profiler (KLA-Tencor) and Atomic Force Microscope (AFM, Bruker, Inc.), as shown in Figures S1(a) and (b) in the Supporting Information, respectively.

### Optical measurement

The *in situ* UV-Vis absorption and the fluorescence emission spectra of the SO-doped PMMA film were obtained with a UV-Vis spectrophotometer and a fiber spectrophotometer (Ocean Optics), respectively.

**Figure 1:**
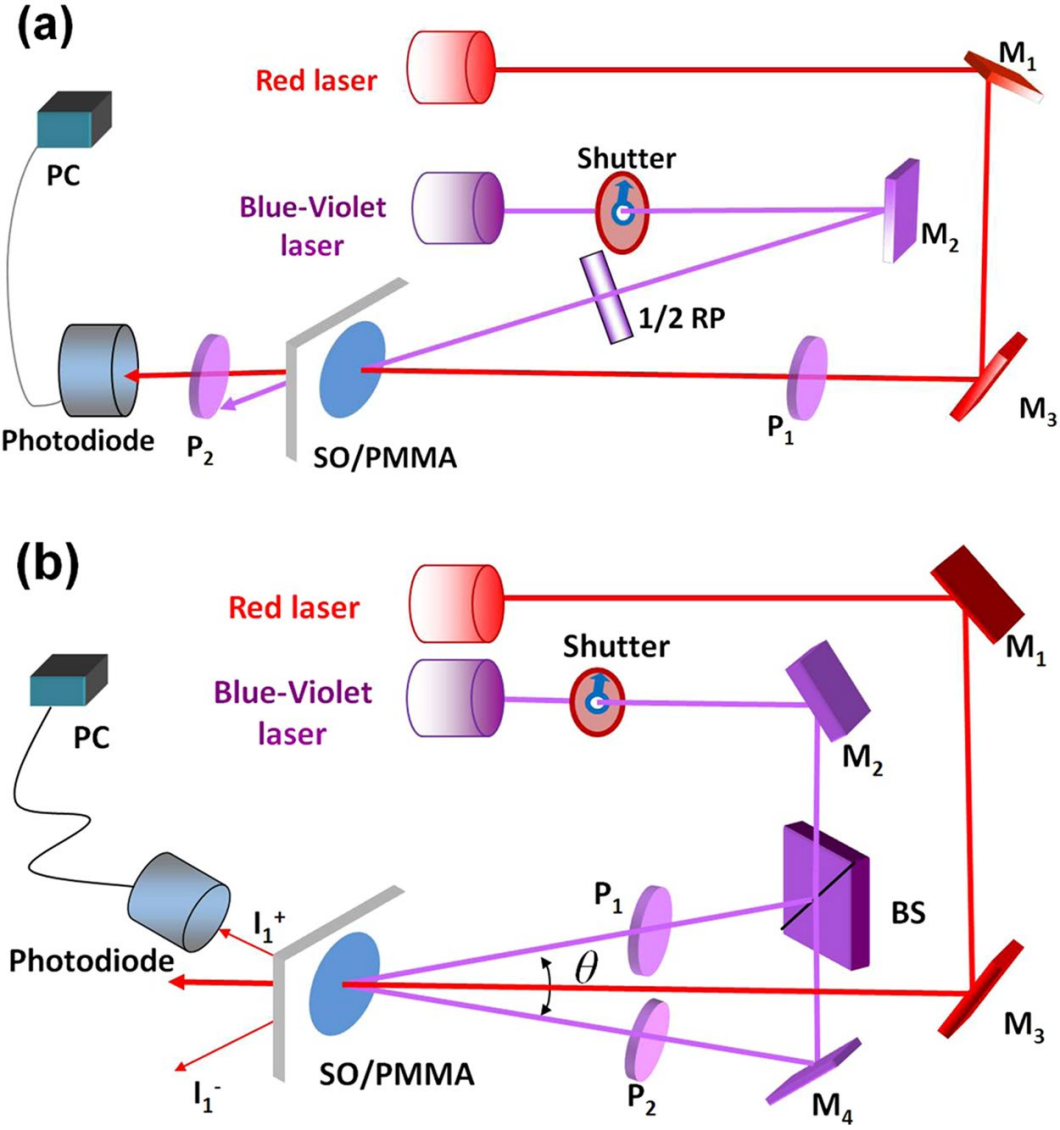
Experimental setups for (**a**) measurement of photoinduced birefringence and (**b**) holographic grating recordings in spirooxazine-doped PMMA films. BS, beam splitter; M, mirror; P, polarizer; RP, retardation plate.

Optical setup for photoinduced birefringence is shown in Fig. 1(a). Two laser beams were nearly parallel incident to the same position on the sample surface. The major elements of the experimental apparatus were a blue-violet laser (TOPTICA Photonics, 403.4 nm) as pumping source and an S-polarized red laser providing probe beam of 671 nm. A half-wave plate was rotated to a proper direction to adjust the polarization state of the pumping beam. The sample was fixed between two crossed polarizers in the path of the probe beam. It was designed to measure the effective phase retardation. The power density of the 671 nm beam was set as 1.42 mW/cm^2^ to reduce the destructive readout. The intersection angle between the polarization states of the pumping and probing beams was 45°. The transmittance of probe beams was monitored by a silicon photodiode interfaced with a computer.

Mono-color holographic grating recording with different writing power densities were carried out by the irradiation of two coherent S-polarized lights from the blue-violet laser on the center of the fixed photochromic film, as shown Fig. 1(b). The intersection angle between the two blue-violet beams was fixed at θ = 6.5°. The grating period can be calculated to be 3.56 μm by the Bragg’s law of Λ = λ/2sin (θ/2), where λ represents the wavelength of the writing beams. In addition, dynamics of holographic grating was monitored using a red laser (671 nm, 2.85 mW/cm^2^, S-polarized). The probe beam was normal incident to the same spot with the writing-beams. The first-order diffraction signal was measured as a function of time by a silicon photodiode interfaced with a computer.

An inverted Confocal Laser Scanning Microscope (CLSM) was used (FluoView FV1000, Olympus, Tokyo, Japan) to observe holographic gratings, in bright and dark field channels, respectively. A 20-mW 488-nm laser (Cyan OEM, Spectra Physics) was chosen as the exciting beam. Surface relief structures for the fluorescent gratings were measured by AFM.

## Results and Discussion

### Formation of Merocyanine Aggregates

**Figure 2:**
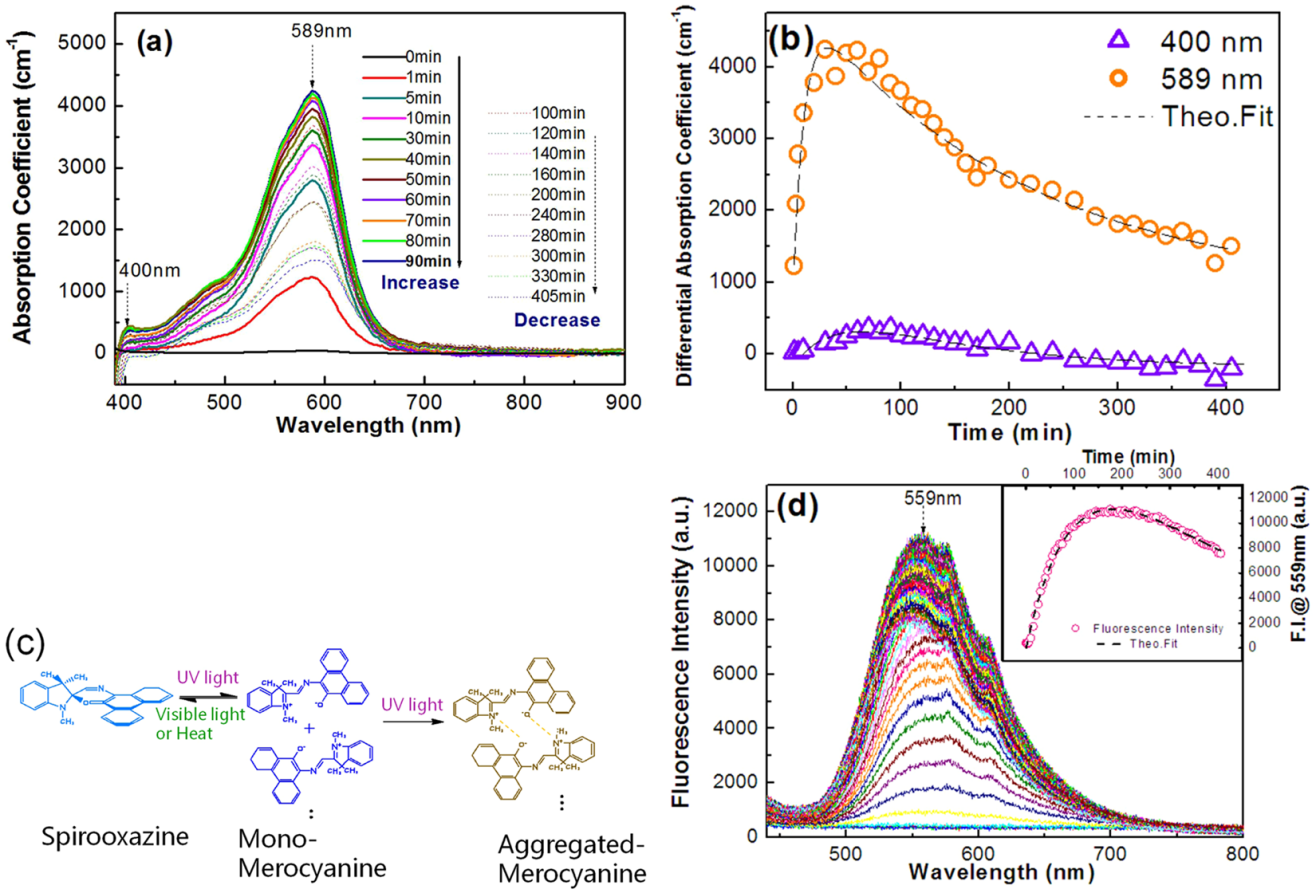
(**a**) Differential absorption spectra in the UV-Vis-NIR region (350–900 nm) of the SO/PMMA film before (black solid line) and after irradiation with linearly polarized light from a blue-violet laser (403.4 nm, 57 mW/cm^2^) for different periods (colorful solid and dash lines). (**b**)Time courses of the absorption peaks (400 nm and 589 nm) under the near-UV excitation for different periods. (**c**) Schematic of the reversible isomerization between spirooxazine (SO) and merocyanine (MC) with different forms. (**d**) The fluorescence intensity of SO/PMMA films irradiated with different irradiation times by mono-color blue-violet beam (403.4 nm, 71 mW/cm^2^). The insert graph presents the corresponding fluorescence intensity at 559 nm versus irradiation time.

The absorption spectra of SO/chloroform solution and SO/PMMA solid film were measured, as shown Figure S2 in the Supporting Information. However, little shift for the absorption peak position was found, indicating that there is no dye aggregation during sample preparation. Figure 2(a) shows that the differential absorption spectra in the UV-Vis region from 350 nm to 900 nm for the SO-doped polymer, excited with the blue-violet laser (57 mW/cm^2^) for different times. Obvious absorbance accumulation appears around 589 nm and another absorption band emerges at ~400 nm under the long-time excitation. Figure 2(b) presents the excitation time dependence of visible absorbance at 400 nm and 589 nm. It is found that the absorption band in the yellow-green region can be enhanced by increasing the irradiation time until 90 min, and then presents gradual decrease. The near-UV absorbance has a similar temporal behavior to the former in the long-time excitation.

The decrease of absorption coefficients at 400 nm and 589 nm in the later excitation stage may be related to photo/thermal bleach and the secondary photo-chemical reaction. The transparent SO molecules are transformed to open ion-form by the C-O bond cleavage under the blue-violet light excitation, resulting in the formation of mono-MC molecules (MC_mono_). The continuous increase of MC_mono_ molecule population results in the enhancement of the absorbance at 589 nm at the initial stage. Meanwhile, the excited molecules can either be reverted to the colorless state thermally (Figure S3a in the Supporting Information) or by visible excitation (Figure S3b in the Supporting Information), or tend to be gathered as MC_agg_, resulting in the later decline of absorbance at 589 nm. Here, the blue-shift of the absorption peak from 589 nm to 400 nm indicates the formation of H-aggregation^17,35,36^. That means MC_mono_ consumption, partly, contributes to the formation of MC_agg_. Chemical structures for SO, mono- and aggregate-forms of MC, and their inter-conversion are all shown in Fig. 2(c). *In situ* fluorescent spectra under the blue-violet excitation of 71.4 mW/cm^2^ for different times were carried out, as show in Fig. 2(d). The fluorescence intensity centered at 559 nm is enhanced by almost 30 fold at the irradiation time of 180 min and then decreases gradually. Under the near-UV excitation, the MC_agg_ with high energy level can release the luminous energy in visible region^37^, which, instead, induces the transformation from MC to SO and weakens the stability of MC_agg_, i.e., fluorescence quenching. Thus the declines both for the absorbance at ~400 nm and the fluorescence intensity at ~559 nm can be explained by the negative role of fluorescence after the long-time excitation.

The generation of carbon dioxide in the formation of merocyanine aggregates was *in situ* monitored using a gas chromatograph (SP-2100A, BFRL Co.). After the near-UV excitation (403.4 nm, 45 mW/cm^2^) on the sample for 420 min, very small amount of carbon dioxide gas, only 13 ppm, was detected, indicating photodegradation process in our case can be ignored.

### Photo-anisotropy by blue-violet excitation

**Figure 3:**
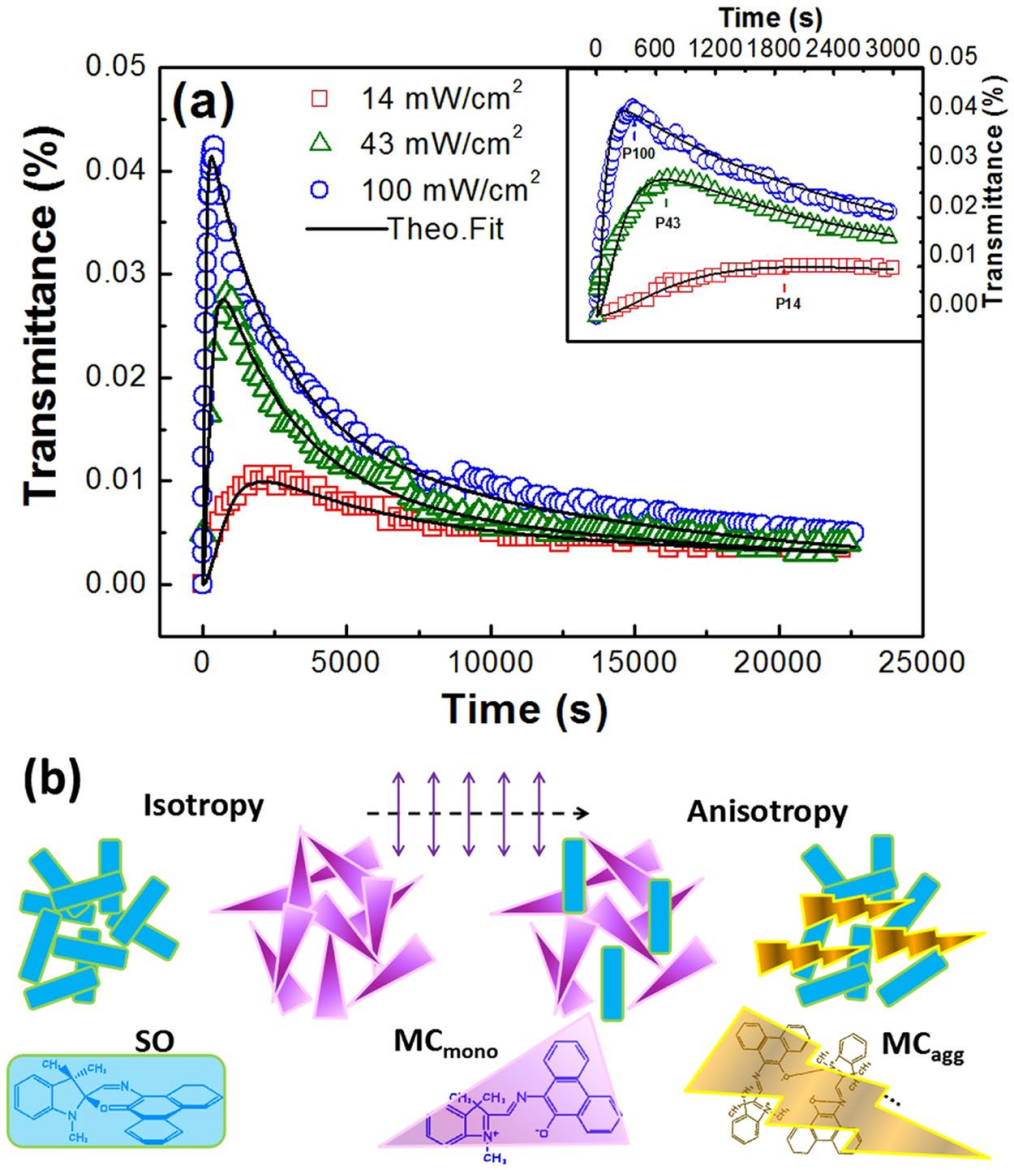
(**a**) Temporal-evolution of photoinduced anisotropy under the +45° polarized co-irradiation of blue-violet laser with different pumping power densities (14, 43 and 100 mW/cm^2^). The solid lines are the theoretical fittings. The inset graph shows the photoinduced anisotropy of the photochromic film at the initial stage (0–3000s). (**b**) Sketch for selective transformation process of SO-MC system versus time under the linearly polarized excitation of 403.4 nm.

Figure 3(a) presents the probe-transmittance versus time for the red light passing through two orthogonal polarizers (0°and 90°), under +45°polarized blue-violet irradiation with different pumping power densities. The transmitted signal, which is related to photo-anisotropy of the film, increases to the maximum value rapidly and decreases gradually versus pumping time. Evidently, the maximum values and the response rates of the transmittance can both be enhanced when increasing the power density of the 403.4 nm light. The transmittance peaks for different pumping power densities (14 mW/cm^2^, 43 mW/cm^2^ and 100 mW/cm^2^) are indicated as P14, P43 and P100 in the inset of Fig. 3(a). After these peaks, the transmitted intensities for different pumping power densities begin to drop gradually and almost reach the same value at ~22000s.

The photo-anisotropy of the SO/PMMA film at the early stage of the pumping process can be explained as the principle of Angular Hole Burning (AHB)^38^, as shown in Fig. 3(b). Because of the insensitivity of SO molecule to the laser polarization state, the near-UV excitation may provoke the ring-opened reaction to produce a large population of the colored molecules in various orientations. The MC molecules with ion form are highly sensitive to linearly-polarized visible irradiation. Along the direction of electronic vector of optical field, positive and negative charge centers of MC are easier to be recovered to the original state, which can be proved by near-UV assisted dichroism experiments (see Figure S4 in the Supporting Information). In fact, due to the location of 403.4 nm at the edge of the visible region, the inverse excitation by blue-violet light may also act on the MC molecules of which the electric dipole distance lies in the direction of the laser polarization state [see Figure S5(a) and (b) in the Supporting Information]. The dual functions of the 403.4 nm excitation result in complex temporal behavior of the anisotropy of the SO-MC system. The long-term stimulation of the near-UV light causes the population decrease of MC molecules which also weakens the film anisotropy. The subsequent formation of H-aggregates of MC presents a head-to-tail structure that the molecular dipoles are arranged in anti-parallel^17^. Thus the photo-induced anisotropy from the formation of MC_agg_ is not strong but stable which contributes to the final transmitted signal.

As the temporal evolution of 671 nm transmittance is similar to the curves of exponential growth with decay term, a dynamics model to explain the photoinduced anisotropy observed in Fig. 3(a) can be expressed as,

1 $${\rm{T}}={\sin }^{2}[{\pi }{\rm{d}}/{\lambda }{\rm{\Delta }}{{n}}_{{\rm{MC}}}({t})]={\sin }^{2}\{{{\rm{\Sigma }}}_{{\rm{i}}}({\pi }{\rm{d}}/{\lambda }){{\rm{\Delta }}n}_{{\rm{i}}}[1-\exp (-{\rm{t}}/{{\rm{\tau }}}_{{\rm{i}}})]\exp (-{t}/{{\rm{\tau }}}_{{\rm{D}},{i}})\},$$

where Δ*n*_i_ (i = momo and agg) is the maximum value of refractive index change resulted from the AHB effect of MC_mono_ and the regular arrangement of MC_agg_, respectively. *τ*_i_ (i = momo and agg) is the anisotropy-formation time constant. *τ*_D,i_ (i = momo and agg) is anisotropy decay time constant. Using this model, theoretical fitting to the experimental curves in Fig. 3(a) for different pumping power densities of 14, 43 and 100 mW/cm^2^ was carried out [see Figure S6(a),(b) and (c), respectively, in the Supporting Information]. The parameter values of the photo-anisotropy experiment (Δ*n*_i_, *τ*_i_ and *τ*_D,i_, i = momo and agg) for different blue-violet laser power densities were determined accordingly, as listed in Table S1 in the Supporting Information. The theoretical fitting results indicate that the AHB effect of MC_mono_ plays a major role in the whole process and contributes to the rapid process of photoinduced anisotropy, while the contribution of MC_agg_ is much weaker and takes effect at the later stage.

### Mono-color Holographic Dynamics

**Figure 4:**
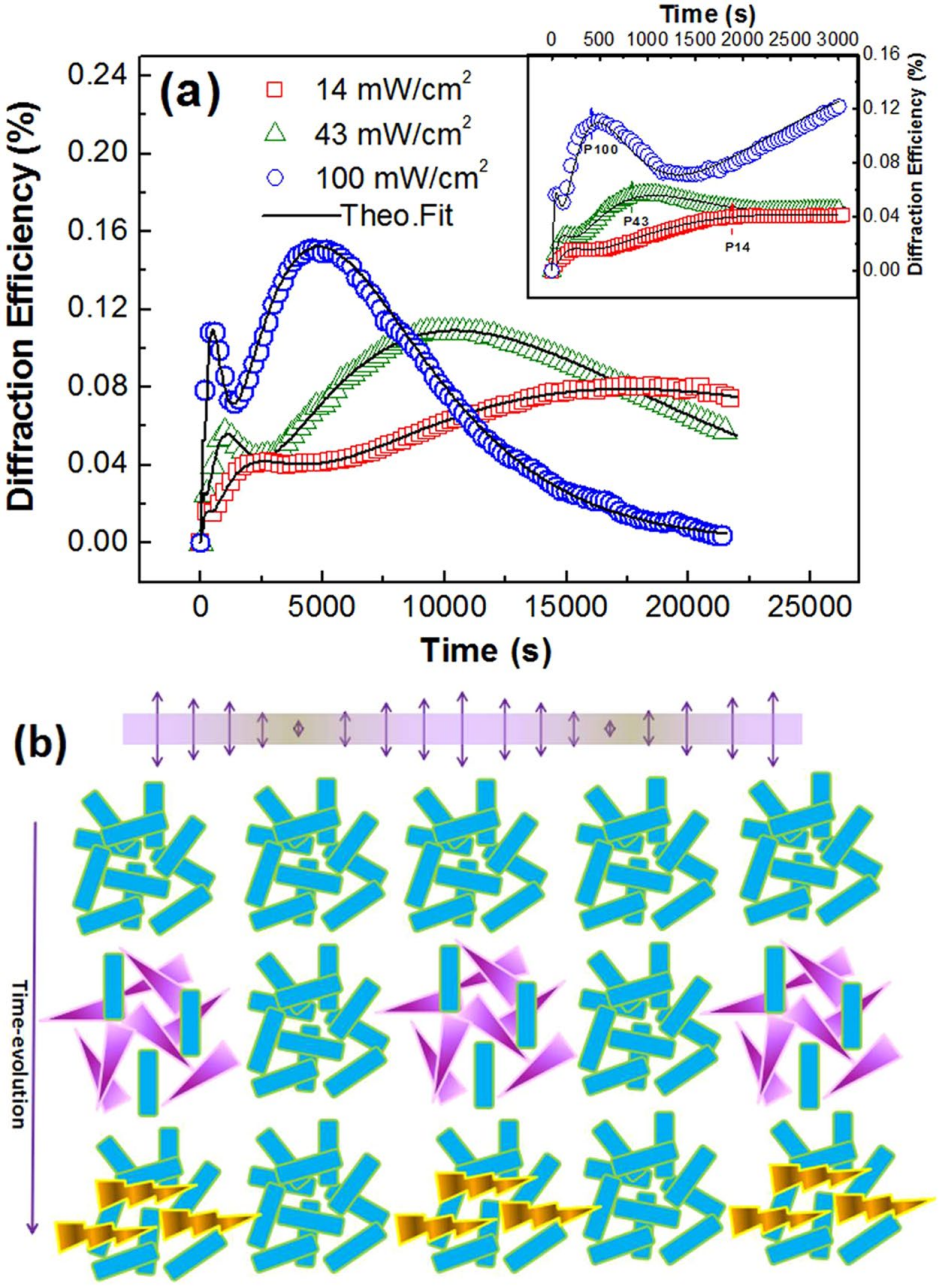
(**a**) Time dependence of the first-order diffraction efficiency in the SO/PMMA film with different writing power densities. The inset graph presents the initial stage (0–3000s) of the grating growth. The solid lines are the theoretical fittings. (**b**) Sketch for time evolution of periodic distributions of the photochromic molecules during the coherent 403.4 nm irradiations.

Due to the stable green emission of MC_agg_, fluorescent micro-patterns were formed via holographic recording. Figure 4(a) shows the temporal evolution of the first-order diffractive intensity of the holographic grating inscribed by the mono-color laser with different power densities of 14, 43 and 100 mW/cm^2^. Clear oscillations of diffractive signal for all the laser power densities appear. Three maxima were observed in the whole holographic kinetics. The diffraction efficiency increases sharply to the first maximum in the initial stage (0~500s) followed by a slight decrease, and then climbs to the secondary high value before 3000s. The third one appears for a rather long recording time with the highest diffraction efficiency. After this point, the intensity of the diffractive signal begins to decrease and tends to be stable.

Based on analysis of the temporal evolution of the near-UV light induced anisotropy, the photo-transformation process of spirooxazine molecules in the coherent linearly-polarized blue-violet excitation can also be divided into two stages: (i) the increase of MC_mono_ molecules and (ii) their subsequent angle-selective inverse conversion in the bright region of the interference fringes, resulting in the quick formation of absorption gratings (AGs) and the gradually accumulated phase gratings (PGs), respectively, as shown in Fig. 4(b). With the population decline of the excited MC molecules, the AG is weakened, causing the slight decrease of diffraction efficiency in the first 500 s. However, the periodic distribution of anisotropy by the AHB effect of MC_mono_ can be built up gradually which also contributes to the increase of diffraction efficiency. Comparison of the response time at peak position (P14, P43 and P100) between the photoinduced anisotropy and holographic dynamics for different near-UV laser power densities indicates that the second maximum of the diffraction efficiency comes from the formation of PGs, as shown in the inset graph of Fig. 4(a). After the long-time excitation, the alternate distributions of stable MC_agg_ at bright regions and SO at dark regions in the interference fringes are produced to cause the appearance of the last diffractive maxima. As the anisotropy of MC_agg_ is rather low, the last peak is ascribed to the other periodic change of absorption coefficient. *In-situ* transmittance measurement at 671 nm also proves the two increases of absorption coefficient and indicates the sequential formation of MC_mono_ and MC_agg_ [see Figure S5(c) in the Supporting Information]. Finally, the fringe contrast for both AG and PG are weakened by the negative role of the green fluorescence.

### Fluorescent Holographic Dynamics Model

Based on the above analysis of the grating growth, the corresponding grating dynamic model is constructed as follows: *η*(*t*) is defined as diffraction efficiency. It has two components resulting from the diffraction on the PG (*η*_*p*_) and AG (*η*_α_)

2 $${\eta }({t})={{\eta }}_{{p}}({t})+{{\eta }}_{{\alpha }}({t})$$

Taking the thin sinusoidal gratings into account, the temporal evolution of diffraction efficiency can be expressed as the sum of the square of the first-order Bessel function of accumulated phase (ΔΓ) and the square of sinusoidal function of absorption coefficient (Δ*α*), as followed:

3 $${\rm{\eta }}({\rm{t}})={{\rm{J}}}_{{\rm{1}}}^{2}[{\rm{\Delta }}{\rm{\Gamma }}]+{\sin }^{2}[{{\rm{d}}}_{{\rm{equ}}}{\rm{\Delta }}{\rm{\alpha }}({\rm{t}})/2]$$

4 $${\rm{\Delta }}{\rm{\Gamma }}=2{\rm{\pi }}{\rm{\Delta }}d{\rm{\Delta }}n/{\rm{\lambda }},$$

where d_equ_ is the equivalent thickness of the thin film (2.38 μm in the case), λ is the wavelength of the probe beam (671 nm in the case), Δn the refractive index and Δd the thickness modulation. The PG kinetics is the result of the co-action of the angle-selective conversion of MC_mono_, the regular arrangement of MC_agg_ and the possible surface relief modulation. The accumulated phase can be expressed as 2πd_equ_Δ*n*_equ_(*t*)/λ, where Δ*n*_equ_(*t*) is equivalent refractive index change taking the three factors mentioned above into account. The expression is reasonable for there is only one maximum in the process of photoinduced anisotropy. The simplified single-exponential growth process with decay term can be expressed as,

5 $${\rm{\Delta }}{{n}}_{{\rm{equ}}}({t})={\rm{\Delta }}{{n}}_{{\rm{equ}},{\rm{\max }}}[1-\exp (-{\rm{t}}/{{\rm{\tau }}}_{{\rm{equ}}})]\exp (-{\rm{t}}/{{\rm{\tau }}}_{D,\mathrm{equ}}),$$

where τ_equ_ is the equivalent recording time constant of PG and τ_D,equ_ the equivalent erasure time constant of PG. For the sequential formation of MC_mono_ and MC_agg_, the dynamic process of the AG can express by bi-exponential response:

6 $$\begin{array}{c}{\rm{\Delta }}{\rm{\alpha }}({\rm{t}})={{\rm{\Delta }}{\rm{\alpha }}}_{{\rm{mono}},{\rm{\max }}}[1-\exp (-{\rm{t}}/{{\rm{\tau }}}_{{\rm{momo}},{\rm{\alpha }}})]\exp (-{\rm{t}}/{{\rm{\tau }}}_{D{\rm{\alpha }},{\rm{Momno}}})\\ \quad \quad \,\,\,\,\,\,\,+{{\rm{\Delta }}{\rm{\alpha }}}_{{\rm{agg}},{\rm{\max }}}[1-\exp (-{\rm{t}}/{{\rm{\tau }}}_{{\rm{agg}},{\rm{\alpha }}})]\exp (-{\rm{t}}/{{\rm{\tau }}}_{D{\rm{\alpha }},\mathrm{agg}}),\end{array}$$

where Δ*α*_*i,max*_ (i = momo, agg) is absorption coefficient with response time variation, *τ*_*i,α*_ is recording time constant of absorption grating, *τ*_*Dα,i*_ is erasure time constant of absorption grating. Consequently, the total diffraction efficiency dynamics can be expressed as follow:

7 $$\begin{array}{rcl}{\rm{\eta }}({\rm{t}}) & = & {{\rm{J}}}_{{\rm{1}}}^{2}\{(2{\rm{\pi }}d/{\rm{\lambda }}){{\rm{\Delta }}n}_{{\rm{equ}},{\rm{\max }}}[1-\exp (-{\rm{t}}/{{\rm{\tau }}}_{{\rm{equ}}})]\exp (-{\rm{t}}/{{\rm{\tau }}}_{{\rm{D}},{\rm{equ}}})\}\\  &  & +{\sin }^{{\rm{2}}}\{{d{\rm{\Delta }}{\rm{\alpha }}}_{{\rm{mono}},{\rm{\max }}}/2[1-\exp (-{\rm{t}}/{{\rm{\tau }}}_{{\rm{momo}},{\rm{\alpha }}})]\exp (-{\rm{t}}/{{\rm{\tau }}}_{D{\rm{\alpha }},{\rm{mono}}})\\  &  & +{d{\rm{\Delta }}{\rm{\alpha }}}_{{\rm{agg}},{\rm{\max }}}/2[1-\exp (-{\rm{t}}/{{\rm{\tau }}}_{\mathrm{agg},{\rm{\alpha }}})]\exp (-{\rm{t}}/{{\rm{\tau }}}_{D{\rm{\alpha }},{\rm{agg}}})\}\end{array}$$

The solid lines in Fig. 4(a) show the kinetics descriptions according to Eq. (7), which agree well with the experimental results. The approximation parameters for all studied cases are gathered in Table 1. It was found that the amplitudes of the formed AG and PG, i.e., Δ*n*_*equ*,max_, Δα_mono,max_ and Δα_agg,max_ are all enhanced with increasing the writing power densities, while all the time constants for either AG or PG exhibit the opposite behavior depending on the excitation intensity.

**Table 1:** Kinetics Parameters Obtained by Fitting to Holographic Experiments with the Different Writing Power Densities of 14 mW/cm^2^, 43 mW/cm^2^ and 100 mW/cm^2^.

Variable		Writing Power Densities		
		14 mW/cm^2^	43 mW/cm^2^	100 mW/cm^2^
Absorption coefficient with response time variation (μm^−1^)	**Δα**_mono,max_	0.0378	0.0521	0.0697
	**Δα**_agg,max_	0.0756	0.111	0.131
Absorption grating recording time constants (s)	**τ**_momo,α_	280	160	56
	**τ**_agg,α_	20000	15000	7000
Absorption grating erasure time constant (s)	**τ**_Dα, mono_	260	135	60
	**τ**_Dα,agg_	28000	15000	7000
Equivalent refractive index change	**Δn**_equ,max_	0.00323	0.00377	0.00449
Phase grating equivalent recording time constant (s)	**τ**_equ_	3000	1290	500
Phase grating equivalent erasure time constant (s)	**τ**_D,equ_	3000	1400	720

The theoretical fitting indicates the photochromism and anisotropy from SO to MC can both be accelerated by introducing high-power excitation light. The formation of MC_agg_ contributes mainly to the AG growth although consuming rather long time. The excitation light with higher power induces more rapid decline of population of MC_mono_, however, provides better condition for the formation of MC_agg_. Due to the light-scattering of the fluorescence from MC_agg_, destructive interference between different bright regions in the holographic fringes is inevitable. The higher the writing power is, the faster the grating erase is.

It was also noticed that the maximum value of diffractive efficiency was achieved to be ~0.15%, which is still lower than that of other photochromic materials, such as azo^39^. The main reason may come from the limited conversion efficiency of SO to MC, the absorbance (exposure sensitivity) of the film at 403.4 nm, and the non-efficient construction of PGs which was proved by holographic recording with different polarization configurations (see Figure S7 in the Supporting Information).

### Fluorescent Holographic Interference Fringes with a Surface Relief Structure

**Figure 5:**
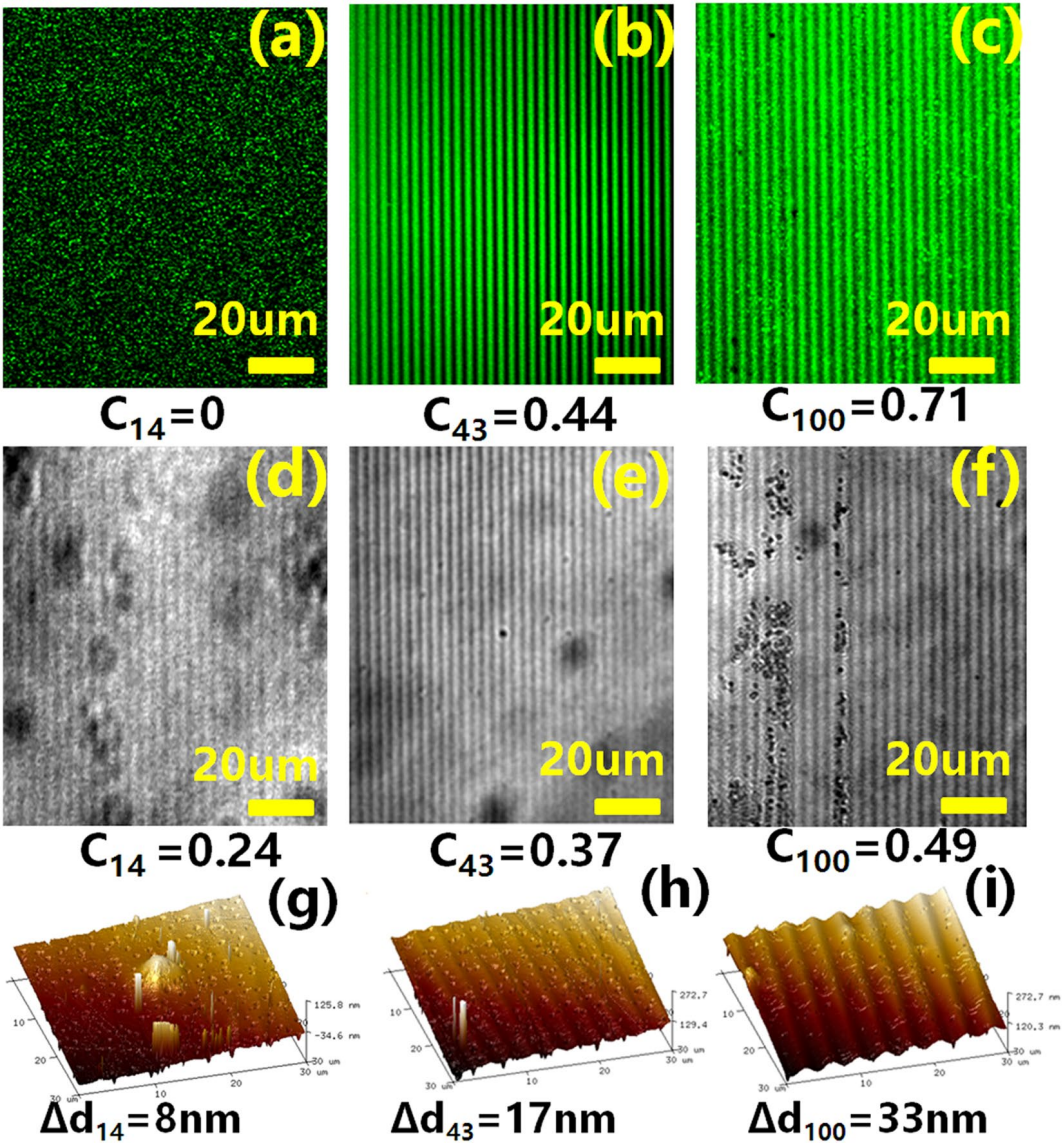
Holographic fringes in the SO-doped polymers observed by CLSM in dark (**a**–**c**) and bright (**d**–**f**) field channels, and by AFM (**g**–**i**) for different writing power densities (14, 43 and 100 mW/cm^2^). The florescent contrast and surface relief modulation of each holographic grating was calculated and labeled.

Under the blue-violet excitation, the stable MC_agg_ provides a possibility for high-efficiency patterned fluorescent emission. CLSM was used to observe, in bright and dark field channels, respectively, the holographic interference fringes in the SO-doped polymer film under different writing power densities. After holographic recording for ~8000s, a 20-mW 488-nm laser (Cyan OEM, Spectra Physics) was chosen as the exciting beam. As shown in Fig. 5(a), almost no periodic structures were observed in the dark field channel after the holographic excitation with the writing power density of 14 mW/cm^2^. However, in the bright field channel (Fig. 5d), holographic gratings were presented clearly. With the increase of the writing power density, the holographic fringes become more clearly in both bright and dark field channels. Surface relief structures for the fluorescent fringes were also measured by AFM, as shown in Fig. 5(g–i). The surface periodic modulation was found to be enhanced versus near-UV excitation intensity accordingly. The fluorescent intensities in the bright and dark regions of the holographic fringes (*I*_max_ and *I*_min_, respectively) were determined by the software attached to the CLSM. The contrast of the holographic fringes (C) was calculated accordingly by the in following formula:

8 $${\rm{C}}=({{I}}_{{\rm{\max }}}-{{I}}_{{\rm{\min }}})/({{I}}_{{\rm{\max }}}+{{I}}_{{\rm{\min }}})$$

The values of *C* were inserted in Fig. 5. Commonly, the variation of Δ*α* is associated with *C* in the bright field channel, while the green fluorescent emission in the dark field channel is related to the formation of MC_agg_. That means the low-power near-UV excitation preferred to construct the alternate arrangement between MC_mono_ and SO. Surface relief modulation (Δd) was also indicated in Fig. 5. The population of MC_agg_ was increased by increasing the irradiation intensity which is beneficial to the formation of surface relief gratings (SRGs). It was also found that the diffraction efficiency of the fluorescent holographic grating can be maintained for a long time after the formation of MC_agg_ (see Figure S8 in the Supporting Information). The results are consistent with the previous analysis of holographic kinetics.

The formation of MC_agg_ plays a role in the non-volatile holographic memory with high-efficiency fluorescent emission and surface relief modulation. The controllable photochemical reaction in SO-MC system also put a bright way to polarization-dependent biomedical detection, and can be applied to the integrated optoelectronic devices with abilities of storage, display and micro-fabrication.

## Conclusions

The photoinduced anisotropy in SO/PMMA films excited by linearly-polarized blue-violet laser beams mainly results from the AHB effect of MC_mono_, while MC_agg_ with antiparallel dipole molecule structure only generates a rather low anisotropic growth but plays an important role in the formation of absorption gratings with the help of long time excitation of coherent lights at 403.4 nm. Taking the measurements of *in situ* absorbance and fluorescence in the photochromic film under the 403.4 nm excitation into account, theoretical description for one phase grating and two sequentially-formed absorption gratings was determined. With the increase of writing light intensity, the amplitudes of absorption and phase gratings were both enhanced; meanwhile the erasure process of the holographic grating was also accelerated. It was found that the writing beams with higher power are helpful to produce MC_agg_ so as to form more stable fluorescence holographic fringe with a surface relief structure, which agrees well with the results of CLSM in bright and dark field channels and AFM observation. The fluorescent periodic structures in blue-violet excitation provide a fabrication strategy for the photon-device with multi-capabilities of information processing.

## Electronic supplementary material

Supporting Information
