# Supplementary material for: Fluorescent Holographic Fringes with a Surface Relief Structure Based on Merocyanine Aggregation Driven by Blue-violet Laser
Source: Sci Rep. 2018 Feb 28;8:3818. doi: 10.1038/s41598-018-22202-2 (PMC5830619; doi:10.1038/s41598-018-22202-2)
Supplement: Supplementary file 1 — Supporting Information [file 41598_2018_22202_MOESM1_ESM.docx]

Supporting Information

**Fluorescent Holographic Fringes with a Surface Relief Structure Based on Merocyanine Aggregation Driven by Blue-violet Laser**

Ruiya Ji^1^, Shencheng Fu^1,*^, Xintong Zhang^1,*^, Xiuxiu Han^1^, Shuangyan Liu^1^, Xiuli Wang^2^ & Yichun Liu^1,*^

^1^ Center for Advanced Optoelectronic Functional Material Research, Northeast Normal University; and Key Laboratory of UV-Emitting Materials and Technology (Northeast Normal University), Ministry of Education, Changchun 130024, P. R. China.

^2^ School of Life Science, Northeast Normal University, Changchun 130024, P. R. China.

^3^ School of Physics, Northeast Normal University, Changchun 130024, P. R. China

* Correspondence and requests for materials should be addressed to S. Fu (email: [fusc515@nenu.edu.cn](mailto:fusc515@163.com)), X. Zhang (email: [xtzhang@nenu.edu.cn](mailto:xtzhang@nenu.edu.cn)) and Y.Liu (email: [ycliu@nenu.edu.cn](mailto:ycliu@nenu.edu.cn)). Tel./Fax.: +86 43185099772.


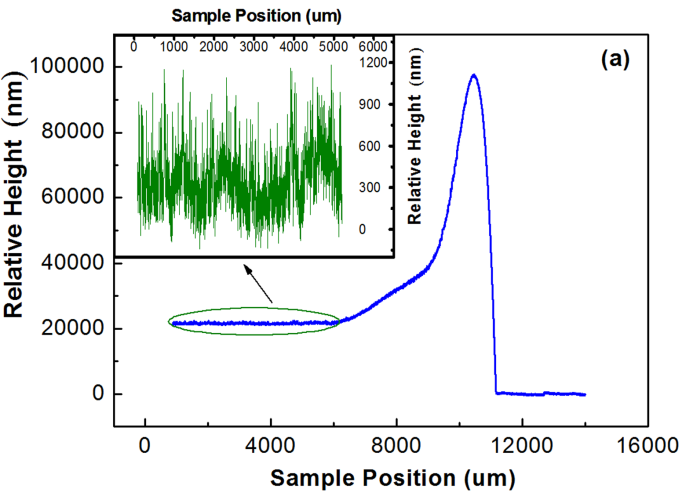

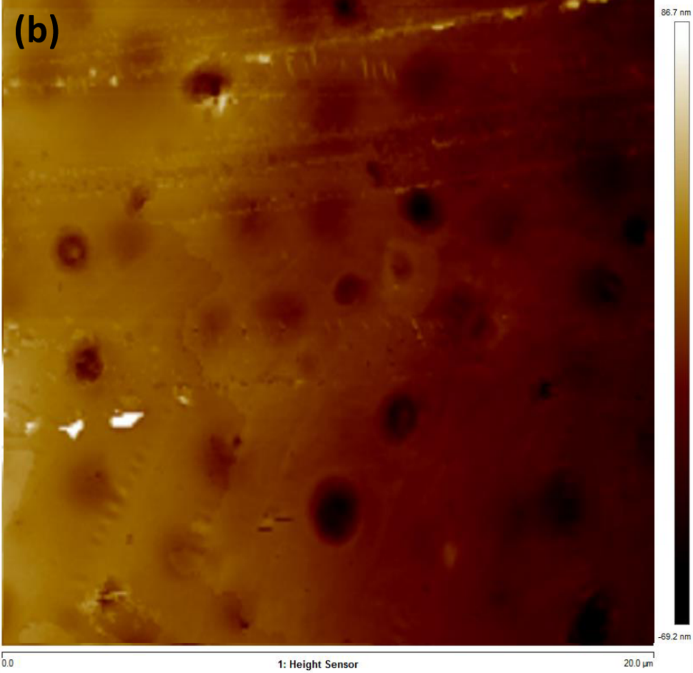


2.38μm

Figure S1 (a) Surface morphology was scanned from center to edge of the sample by a step profiler (KLA-Tencor). (b) The resultant film morphology was observed by the Atomic Force Microscope (AFM, Bruker, Inc.).


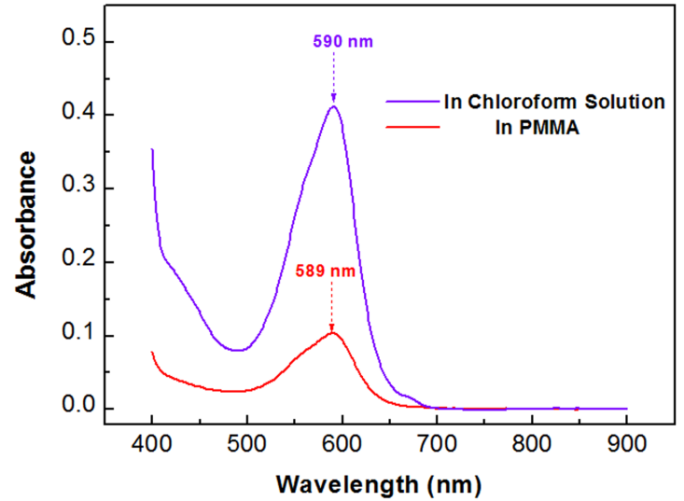


Figure S2 Comparison of the absorption spectra for SO/chloroform solution and the SO/PMMA solid film.


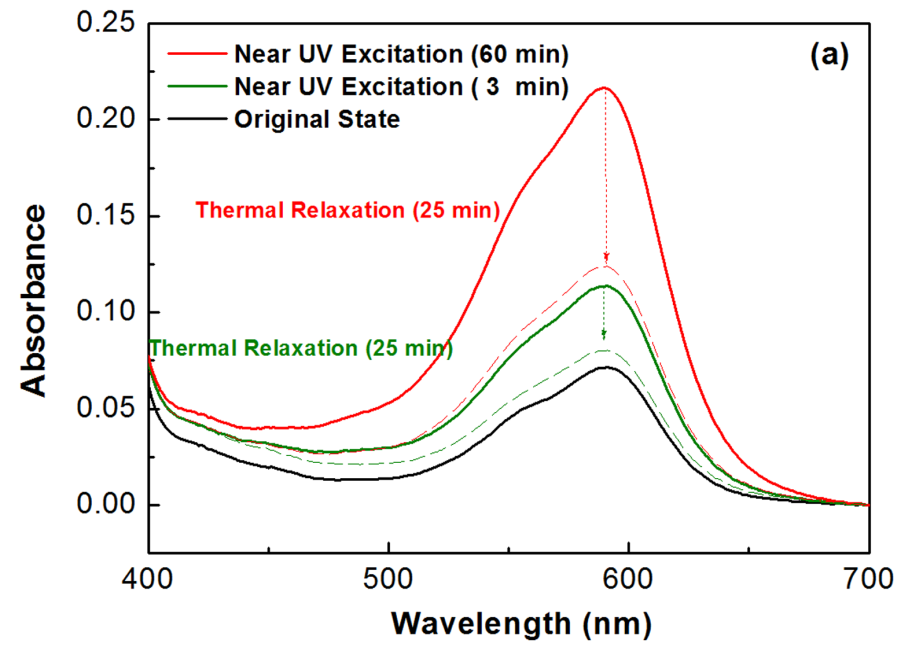


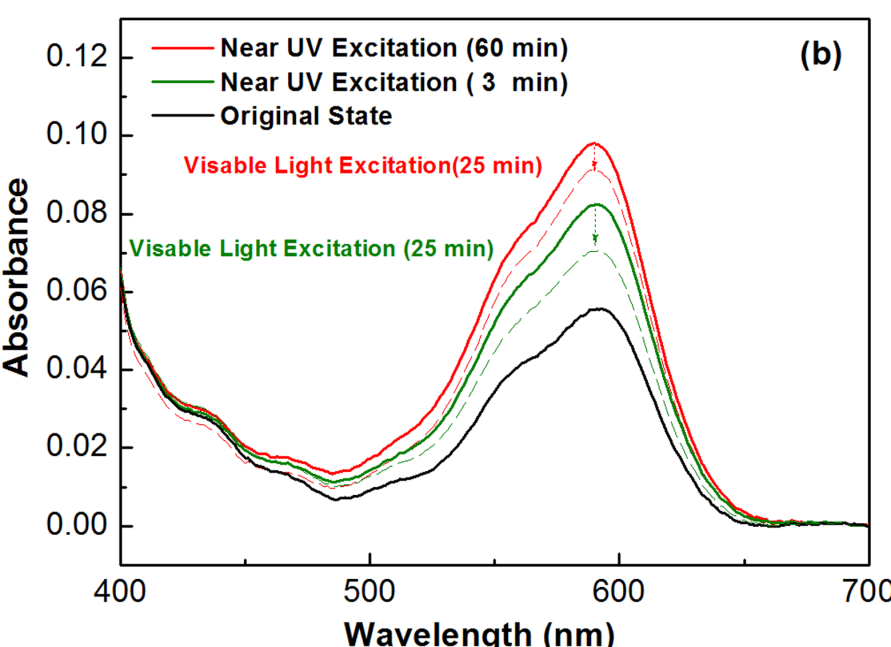


Figure S3 (a) Absorption spectra in the visible region (400−700 nm) of the SO/PMMA film before (black solid line) and after irradiation with linearly polarized light from a blue-violet laser (403.4 nm, 42 mW/cm^2^) for different excitation times (red and green solid lines), and thermal relaxation for 25 min (red and green dash lines). (b) Absorption spectra in the visible region (400−700 nm) of the SO/PMMA film before (black solid line) and after irradiation with linearly polarized light from a blue-violet laser (403.4 nm, 42 mW/cm^2^) for different excitation times (red and green solid lines), and visible light excitation (532 nm, 7.1 mW/cm^2^) for 25 min (red and green dash lines).

Accompanied with circularly polarized near-UV irradiation, spirooxazine (SO) can be converted into the colored form of merocyanine (MC) randomly, resulting in the rapid decrease of transmittance at 532 nm. As shown in Figure S4 (a), the 532 nm beam of 1.4 mW/cm^2^ with S-polarization state is set as probe. The decline of transmittance for P-polarized pumping (71 mW/cm^2^) is faster than that for S-one (71 mW/cm^2^). It is indicated that the transformation from MC to SO by the green laser beam is polarization-dependent, as shown in Figure S4 (b).


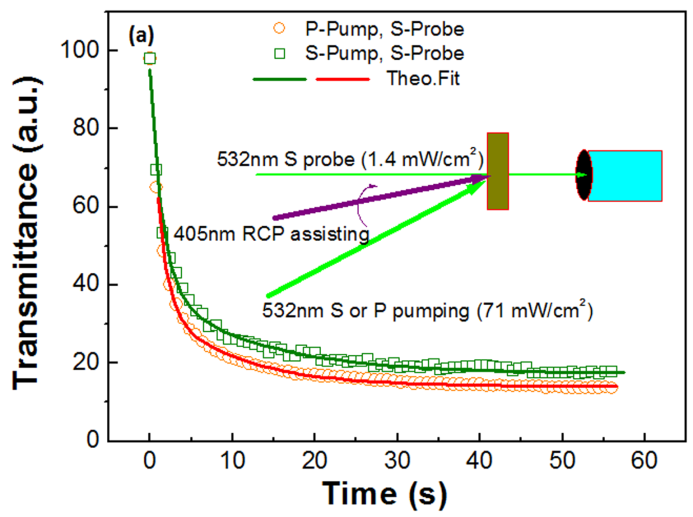


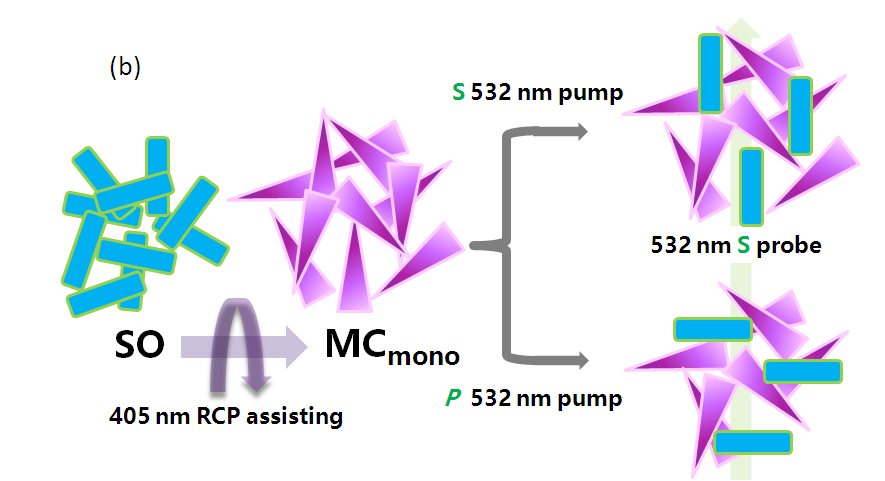


Figure S4 (a) Transmittance at 532 nm versus time in near-UV assisted dichroism measurement. Optical setup is inserted. (b) Schematic diagram of SO-MC molecular transformation under the green excitation with different polarization states (S and P).


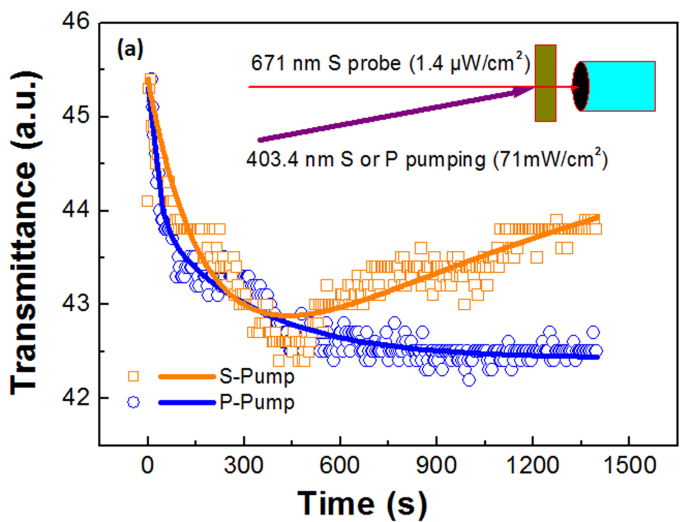


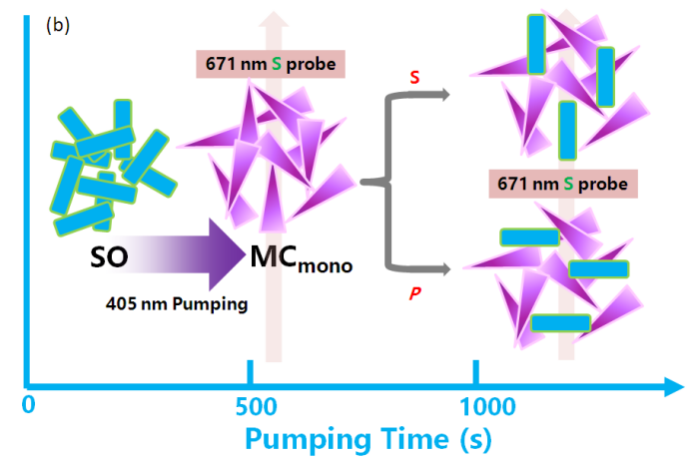


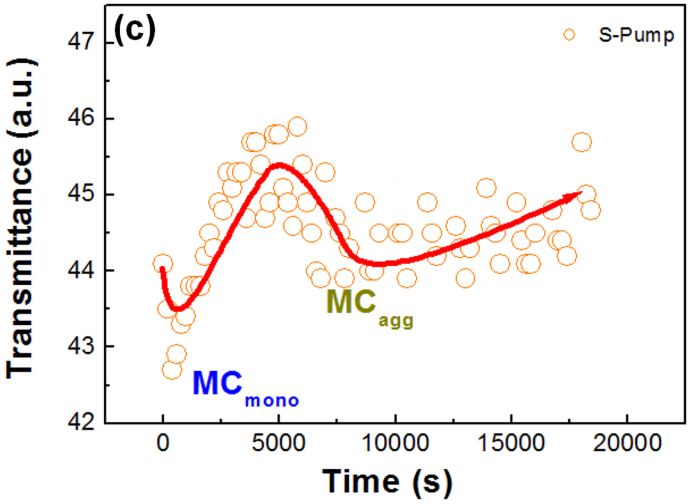


Figure S5 (a) Transmittance at 671 nm versus time in near-UV pumping dichroism measurement. Optical setup is inserted. In the stage of 0-300 s, the near-UV light transforms SO to MC dominantly. After that point, the reverse conversion with polarization-sensitivity plays a major role, resulting in the increase of transmittance under S-polarized excitation; while the value of transmittance is maintained under P one even after further excitation. (b) Schematic diagram of SO-MC molecular transformation under the green excitation with different polarization states to explain the phenomenon of Figure S5 (a). (c) Transmittance at 671 nm versus time in near-UV pumping dichroism measurement prolonging to 18000 s. Two minima correspond to the effective formation of MC_mono_ and MC_agg_, respectively.


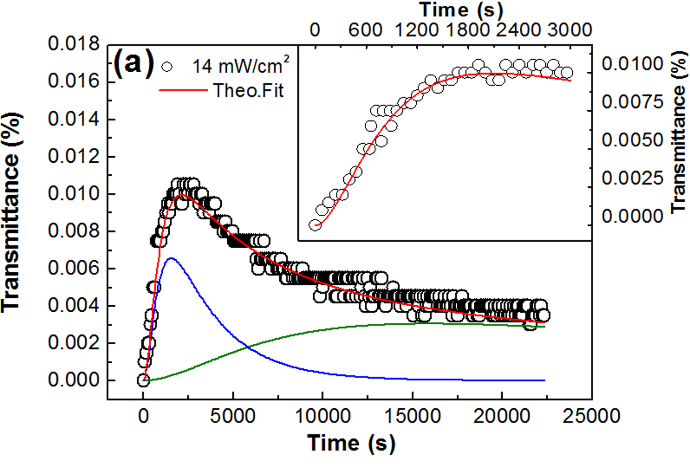


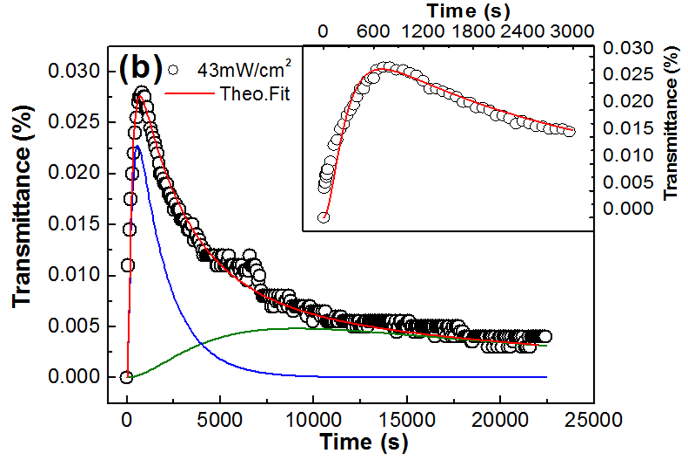


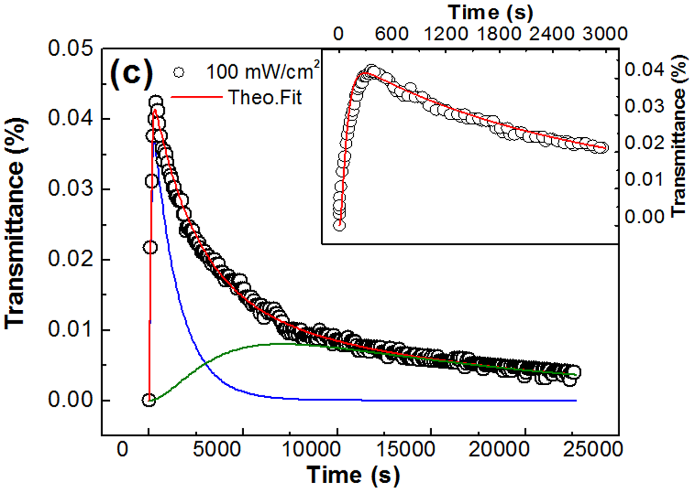


Figure S6 (a), (b) and (c) Photoinduced anisotropy under the 45°-polarized co-irradiation of blue-violet laser with power densities of 14, 43 and 100 mW/cm^2^, respectively. The kinetics is fitted by Eq. (S1). The insert graphs present the initial stage of photoinduced anisotropy process. The dash lines are the simulation of fast (blue) and slow (green) processes of photoinduced anisotropy, resulting from Angular Hole Burning effect of MC_mono_ and regular alignment of MC_agg_, respectively.

T=sin^2^[*πd/λΔn_MC_(t)*]= sin^2^{(*πd/λ)Δn_mono_*[1-exp(-t/τ_mono_)] exp(-*t/*τ*_D,mono_*) +(*πd/λ)Δn_agg_*[1-exp(-t/τ_agg_)] exp(-*t/*τ*_D,agg_*)} (S1)

According to Eq. (S1), theoretical fitting parameters are gathered in Table S1.

Table S1 Approximation Parameters Obtained by Fitting to the Photoinduced Anisotropy Curves for the Pumping Power Densities of 14 mW/cm^2^, 43 mW/cm^2^ and 100 mW/cm^2^.

|  | | Writing Power Densities | | |  |
| --- | --- | --- | --- | --- | --- |
| Variable | | 14mW/cm^2^ | 43mW/cm^2^ | 100mW/cm^2^ | |
| Maximum refractive index | Δn_mono,max_ | 0.00108 | 0.00171 | 0.00193 | |
|  | Δn_agg, max_ | 0.000629 | 0.000808 | 0.00108 |  |
| Anisotropy formation time constant (s) | τ_momo,α_ | 700 | 200 | 62 |  |
|  | τ_agg,α_ | 5600 | 3500 | 2750 |  |
| Anisotropy decay time constant (s) | τ_Dα,Momno_ | 5600 | 3350 | 2750 |  |
|  | τ_Dα,agg_ | 90000 | 47000 | 33000 |  |


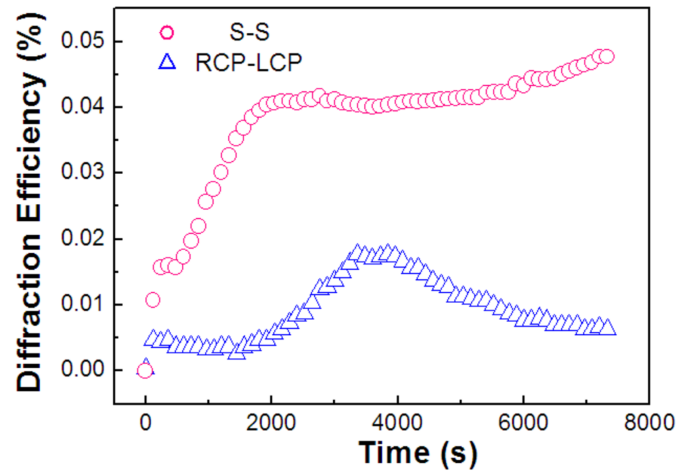


Figure S7 Temporal evolution of diffraction efficiency for different polarization configurations of (S-S) and (RCP-LCP) in spirooxazine-doped polymers.


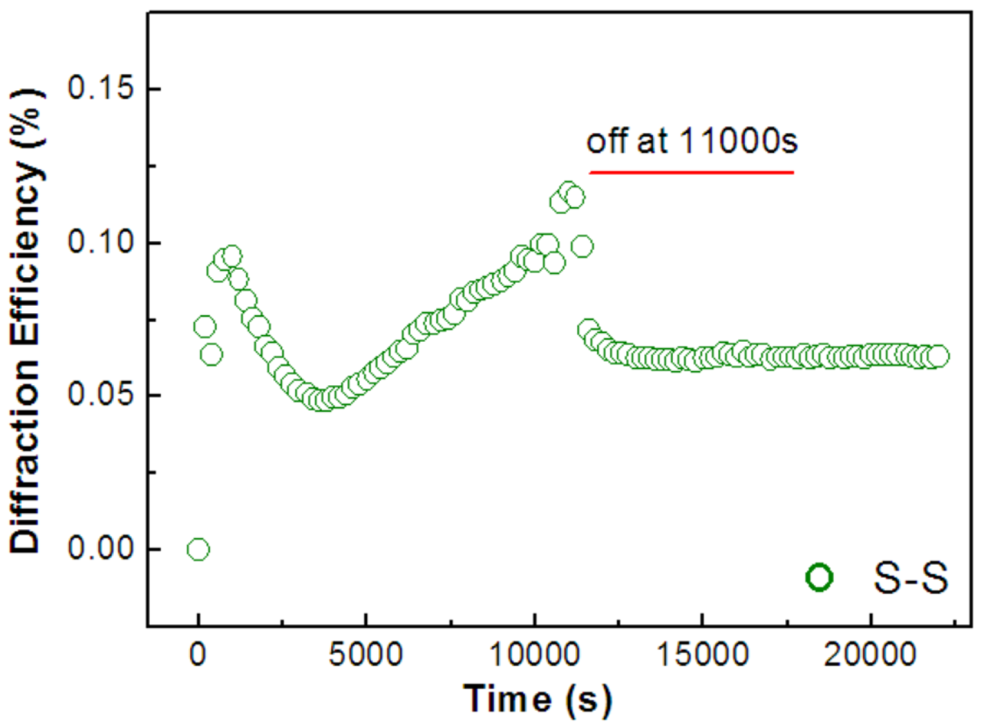


Figure S8 First-order diffraction efficiency of holographic gratings in the SO/PMMA film under the (S-S) recording. At 11000 s, the writing lights were turned off. It was found that the diffraction efficiency of the grating decays ~30 % and keeps constant to 22000 s.
